# Dynamic changes in gut microbiota and metabolites in advanced lung cancer patients with immune-related adverse events

**DOI:** 10.3389/fimmu.2026.1731931

**Published:** 2026-04-16

**Authors:** Xu Han, Dan Zang, Meixi Lin, Yuting Yin, Dan Liu, Qiangguo Sun, Jun Chen

**Affiliations:** 1 Department of Oncology, The Second Hospital of Dalian Medical University, Dalian, China; 2 Department of Respiratory, The Second Hospital of Dalian Medical University, Dalian, China

**Keywords:** dynamic changes, gut metabolites, gut microbiota, immune-related adverse events, lung cancer

## Abstract

**Background:**

Immune-related adverse events (irAEs) represent an urgent clinical challenge. Although accumulating evidence suggests that irAEs are associated with the gut microbiota and its metabolites, our understanding of the dynamic alterations in the gut microbiota and related metabolic profiles throughout the onset and progression of irAEs remains limited.

**Methods:**

A total of 48 fecal samples were collected from 32 lung cancer patients treated with immune checkpoint inhibitors, including 16 patients who developed irAEs and 16 who did not. Fecal samples were collected at baseline and, in patients with irAEs, at the time of irAEs onset. Metagenomic sequencing and untargeted metabolomics analyses were performed to identify baseline differences in gut microbiota and metabolites, characterize longitudinal dynamic changes in gut microbiota and metabolite profiles in patients with irAEs, and construct a machine learning based random forest model to predict the occurrence of irAEs.

**Results:**

There were baseline differences in microbial communities and metabolites between the two groups. In the non-irAEs group, *Phocaeicola coprocola* was enriched and *Micrococales* decreased. At baseline, viomycin was positively correlated with irAEs, while metabolites such as calcitriol and L-isoleucine were negatively correlated with irAEs. The roles of valine, leucine and isoleucine metabolism and vitamin B6 metabolism pathways were downregulated in the irAEs group. Compared to baseline, there were significant changes in gut microbiota and metabolites during the onset of irAEs, and the abundance of *Veillonella* increased during irAEs onset. Dynamic monitoring of metabolic changes in irAEs revealed decreased levels of trypsin butylester, BQ 123, DL-o-tyrosine, and nicotinamide-beta-riboside during irAEs attacks. Lysine degradation, arachidonic acid metabolism, folate biosynthesis, nicotinate and nicotinamide metabolism, and C5-branched dibasic acid metabolism were downregulated during the progression of irAEs. A model for predicting the occurrence of irAEs based on differential microbiota and metabolites was constructed, and after robust validation, the model showed good performance and excellent discriminative power.

**Conclusions:**

The occurrence and development of irAEs are associated with the composition of the gut microbiota and metabolites, as well as their dynamic changes over time. These findings highlight the potential of gut microbiota and metabolites as biomarkers for predicting the occurrence and progression of irAEs.

## 1 Background

Immune checkpoint inhibitors (ICIs) are among the most important treatments for advanced lung cancer, but immune-related adverse events (irAEs) pose significant challenges to the effectiveness and safety of ICIs (1). When ICIs excessively activate the immune system and trigger immune attacks against normal tissues, irAEs can occur, and these autoimmune toxicities may involve almost any organ system. Among cancer patients receiving ICIs, lung cancer has been reported to have the highest incidence of irAEs and a high burden of severe grade irAEs. One study analyzing cancer patients who experienced irAEs found that 32.5%-87% were lung cancer patients, and that lung cancer patients accounted for 34.2%-86.6% of cancer patients with severe grade irAEs (2). The incidence of irAEs in non-small cell lung cancer is 37% -48% (3–5), and in small cell lung cancer is 57% (6). Because of irAEs, many patients are forced to discontinue treatment despite deriving clinical benefit from ICIs. Given the high incidence of irAEs in lung cancer patients receiving ICIs, it is essential in clinical practice to balance the risk of irAEs against the potential benefits of ICIs before treatment initiation. However, there is currently no effective approach to provide early warning of irAEs occurrence. Therefore, developing potential biomarkers to predict irAEs is necessary to identify patients at high risk of irAEs and enable early intervention (7).

As is well known, the human gut microbiota and its metabolites can modulate immune homeostasis and tolerance. Microbial diversity and community composition, together with microbiota driven metabolic effects, may influence irAEs by reshaping immune components within tumors and in the circulation (8–10). The human gut is directly exposed to the external environment, making the composition of the gut microbiota readily influenced by environmental factors and prone to change. At the same time, the dynamic balance between gut microbiota and metabolites is closely linked to human health and disease, and shifts in gut microbiota over time can influence disease progression (11). Understanding the dynamic changes in gut microbiota during the onset of irAEs is crucial for enabling real-time monitoring of irAEs occurrence during ICI treatment.

In this study, we collected fecal samples from lung cancer patients before and during treatment with ICIs. We performed metagenomic sequencing and untargeted metabolomics analyses to characterize irAEs associated gut microbiota and metabolites, and to delineate the dynamic changes in microbial communities and metabolic profiles during the onset and progression of irAEs, with the aim of developing a model to predict irAEs occurrence in lung cancer patients receiving ICIs treatment. Our findings provide new insights into the prevention and monitoring of irAEs, and suggest potential mechanisms from the perspectives of microbial composition and metabolic function.

## 2 Materials and methods

### 2.1 Study population and sample

This is a retrospective study. Recruit lung cancer patients with a pathological diagnosis at the Second Hospital of Dalian Medical University, all of whom received immunotherapy alone or in combination (**Supplementary Figure 1**). The included patients met the following criteria: (i) receiving PD-1/PD-L1 inhibitor treatment; (ii) Lung cancer confirmed by histology or cytology; (iii) Complete ≥ 4 cycles of ICIs treatment or irAEs occurrence. Patients who met any of the following criteria were excluded: (i) exposure to antibiotics, probiotics, or high-dose corticosteroids within the 4 weeks prior to enrollment; (ii) Antibiotics, probiotics, or high-dose corticosteroids are required during the follow-up period; (iii) Patients with autoimmune diseases or gastrointestinal diseases. PD-1/PD-L1 inhibitors were administered intravenously every 3 weeks at the recommended dosage, and treatment was terminated when disease progression or intolerable toxicity was observed. This study included a total of 32 patients, including 16 patients without irAEs and 16 patients who stopped receiving immunotherapy due to irAEs. Baseline fecal samples were collected from all patients before ICIs treatment, and patients who experienced irAEs were required to collect fecal samples during the onset of irAEs. After sample collection, samples were stored at -80 °C until use.

The study protocol and the informed consent form were approved by the Ethics Review Committee of the Second Hospital of Dalian Medical University (Ethics Approval No. 2022‐173). Written informed consent was obtained from all participants.

### 2.2 Data collection

Clinical data were collected, including age, gender, tumor histological type, clinical stage, treatment plan, anti-PD-1 treatment process, and irAEs type. The outcomes of irAEs were evaluated according to the National Comprehensive Cancer Network (NCCN) guidelines for immunotherapy related toxicity management, and the severity of irAEs was assessed using the Common Terminology Criteria for Adverse Events (V.5.0).

### 2.3 Metagenomics sequencing

Total genomic DNA was extracted from fecal samples using the PowerSoil DNA Isolation kit (Mo Bio Laboratories). DNA quality and quantity were assessed using Qubit 3.0 Fluorometer (Life Technologies) and 1% agarose gel electrophoresis. Paired‐end libraries were prepared using the VAHTS Universal Plus DNA Library Prep Kit (Vazyme Biotech) and sequenced on an Illumina NovaSeq 6000 platform using paired‐end mode. Raw sequence data were quality‐filtered using Trimmomatic v0.33. The human reference genome sequence was downloaded from the NCBI database. After constructing the Bowtie2 index, Bowtie2 alignment was performed to filter out human-derived reads, thereby removing host contamination, enriching microbial sequences, and improving the signal-to-noise ratio and computational efficiency of subsequent microbiome analyses. Use MEGAHIT for metagenomic assembly to obtain contig sequences for each sample’s metagenomic assembly. Based on the assembly results of individual samples, predict the encoding genes and construct a non redundant gene set for subsequent analysis.

### 2.4 Metabolomics

Weigh the fecal sample, add an appropriate volume of extraction solution (methanol: acetonitrile: water volume ratio=2:2:1) and magnetic beads for grinding and ultrasonic treatment. After standing and centrifuging, take the supernatant for vacuum drying treatment. Then add an appropriate amount of extraction solution for reconstitution and test on the machine. The LC/MS system for metabolomics analysis is composed of Waters Acquity I-Class PLUS ultra-high performance liquid tandem Waters Xevo G2-XS QTof high resolution mass spectrometer. The column used is purchased from Waters Acquity UPLC HSS T3 column (1.8um 2.1*100mm). Metabolites were detected in both negative and positive ion models. The raw data collected using MassLynx V4.2 is processed by Progenesis QI software for peak extraction, peak alignment and other data processing operations, based on the Progenesis QI software online METLIN database and public database for identification, Simultaneously conducting theoretical fragment identification. After qualitative and quantitative analysis of metabolites, data quality assessment, annotation analysis, differential expression analysis, and functional enrichment are performed.

### 2.5 Data processing and statistical analysis

Perform statistical analysis on clinical data using GraphPad Prism 9.5 and SPSS 25.0. Quantitative variables are represented as Mean ± SD (Mean ± standard deviation) based on their normal distribution. Qualitative variables are represented by numbers and percentages. Compare normally distributed groups using Student’s t-test or one-way ANOVA, and non normally distributed groups using Mann Whitney U test or Kruskal Wallis test. The calculation of rate and composition ratio adopts Pearson chi square test or Fisher’s exact test. Perform correlation analysis using Spearman correlation coefficient. All statistical tests with a two-sided P<0.05 are considered statistically significant.

### 2.6 Bioinformatics data analysis

Use the bray_Curtis algorithm to analyze Beta diversity using distance matrix, and then perform similarity analysis using Permanova. Use Wilcoxon rank sum test to evaluate the differences in species abundance between the two groups. Differences in genera were evaluated by linear discriminant analysis effect size (LEfSe) at the genus level with an Linear Discriminant Analysis (LDA) score threshold of 3. To identify differential microbial taxa associated with irAEs and to validate the LEfSe results, we performed MaAsLin2 analysis. The data were preprocessed using total sum normalization, with irAEs occurrence status and time point included as fixed effects. Relying solely on LEfSe’s default parameters (LDA score) for feature selection may not be sufficient to strictly control for false positives. Therefore, we performed a complementary validation of the MaAsLin2 results by applying the Benjamini-Hochberg method to correct for the false discovery rate (FDR). We set the significance threshold at FDR < 0.05. We selected the top 80 species in terms of abundance and conducted correlation analysis and constructed a correlation network based on the abundance and variation of species in each sample. In the comparisons of metabolite concentrations and other multi-group analyses, p-values were also adjusted using the BH method to control the FDR, and an adjusted p-value < 0.05 was considered statistically significant.

Use databases such as Kyoto Encyclopedia of Genes and Genomes (KEGG), Human Metabolome Database (HMDB), and Lipid Metabolites and Pathway Strategies (LIPID MAPS) to annotate all identified metabolites. Orthogonal partial least squares discriminant analysis (OPLS-DA) was used to compare the main distribution differences between two groups of metabolites. Based on the OPLS-DA results, differential metabolites were screened by combining multivariate analysis of OPLS-DA model’s Variable Importance in Projection with univariate analysis’s p-value or fold change. Perform receiver operating characteristic curve (ROC) analysis separately on the differentially expressed metabolites selected from each group, and calculate the Area under the curve (AUC) results. Annotate differential metabolites using the KEGG database and perform enrichment analysis using hypergeometric tests using the clusterProfiler software package.

In a random forest model, multiple decision trees are constructed using a random subset of training samples and features. Use 10 fold cross validation to evaluate the performance of the random forest model to ensure robustness and prevent overfitting. The importance of variables is calculated based on the average decrease in accuracy and Gini coefficient scores, allowing for the identification of the most representative microbial species and metabolites.

## 3 Results

### 3.1 Demographic and clinical characteristics of the study cohort

This study included 32 patients, including 16 lung cancer patients who developed irAEs after receiving ICIs treatment (irAEs group) and 16 lung cancer patients who did not develop irAEs after receiving ICIs treatment (non-irAEs group). There were no significant differences in demographic characteristics between the irAEs group and the non-irAEs group, including age, gender, pathological type, and tumor staging. These results indicate that the baseline features of the two groups were well balanced and comparable (**Table 1**).

**Table 1:** Demographics and characteristics of the patients.

Characteristic		irAEs patients (n=16)	Non-irAEs patients(n=16)	*P* -value
Age, Mean ± SD years		66.19 ± 10.55	63.31 ± 9.87	0.4168
Sex, *n* (%)	Male	14(87.5%)	16(100%)	0.4839
	Female	2(12.5%)	0(0%)	
Pathological types, *n* (%)	Adenocarcinoma	2(12.5%)	1(6.25%)	0.9999
	Squamous cell carcinoma	9(56.25%)	8(50%)	
	Small cell lung carcinoma	5(31.25%)	7(43.75%)	
Stage	III	6(37.5%)	6(37.5%)	0.9999
	IV	10(62.5%)	10(62.5%)	
Anti-PD-(L)1 types, *n* (%)	PD-1	9(56.25%)	11(68.75%)	0.7016
	PD-L1	7(43.75%)	5(31.25%)	
irAEs types, *n* (%)	Pneumonia	8(50%)		
	Rash	2(12.5%)		
	Hepatitis	3(18.75%)		
	Thyroid dysfunction	2(12.5%)		
	Asthenia	1(6.25%)		

### 3.2 Potential biomarkers of irAEs in baseline gut microbiota

**Figure 1:**
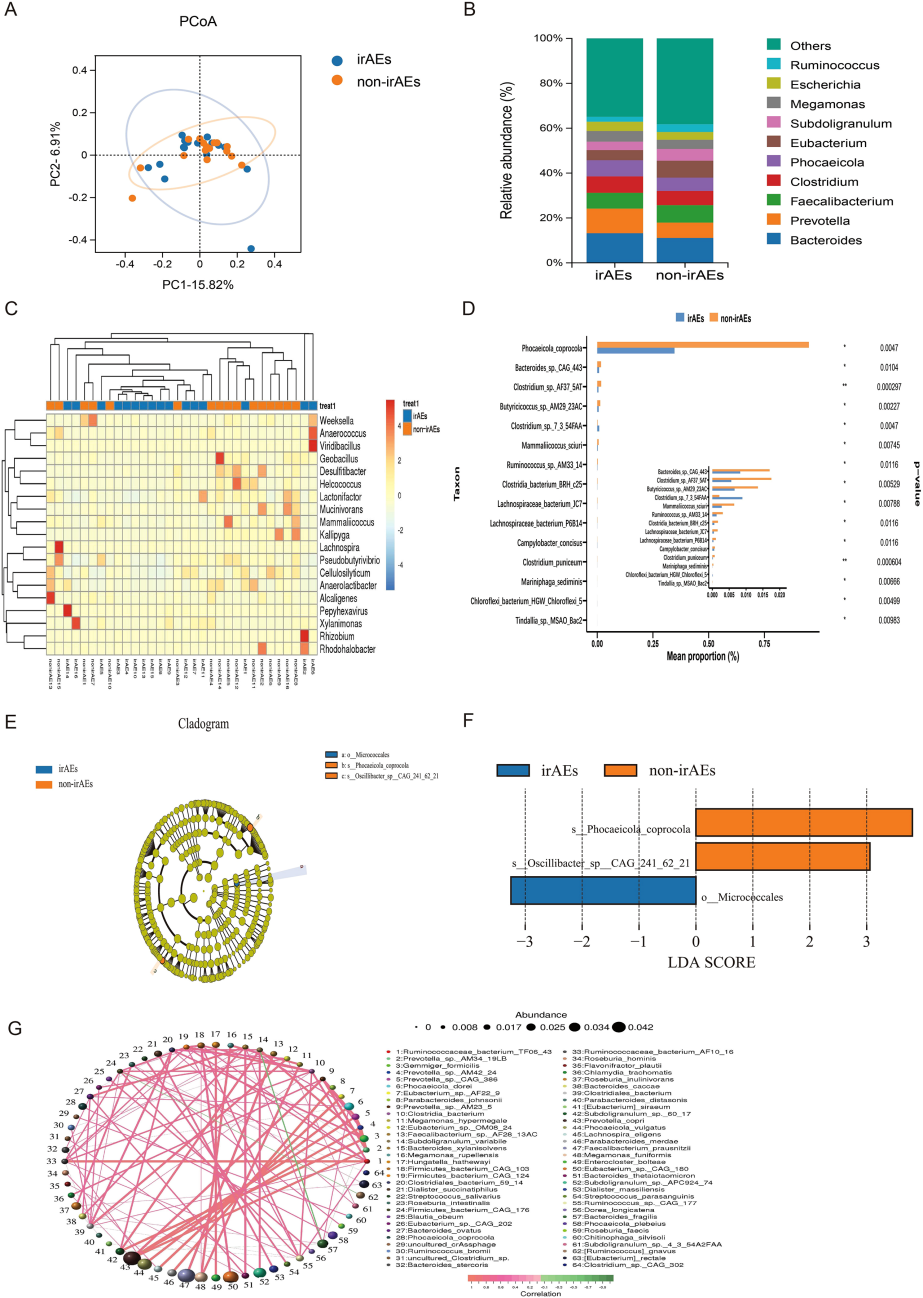
The baseline gut microbiota of patients with/without irAEs. **(A)** Beta diversity analysis. **(B)** Histogram of gut microbiota relative abundance with the top 10 genera. **(C)** Heatmap of differentially abundant genera. **(D)** Bar chart showing the abundance of differential genera. **(E)** Circular cladogram illustrating taxonomic branching patterns. **(F)** Bar chart of LDA scores for discriminative taxa (LDA ≥ 3). **(G)** Species -level co-occurrence network of microbial associations.

We conducted metagenomic analysis on baseline fecal samples. Principal Coordinates Analysis (PCoA) was used to characterize beta diversity, the results showed separation between the two groups, indicating differences in microbial community structure between them (*P* = 0.047) (**Figure 1A**). In the histogram of the top 10 genera, the relative abundance of *Phocaeicola* and *Prevotella* in the irAEs group was higher than that in the non-irAEs group at baseline (**Figure 1B**). The clustering heatmap further demonstrated distinct gut microbiota profiles and dominant bacterial genera between the two groups, warranting further investigation (**Figure 1C**). Inter-group differential species analysis showed that *Phocaeicola coprocola* was enriched in the non-irAEs group (**Figure 1D**). To further identify differential taxa, we performed LEfSe analysis, which revealed group-specific gut microbiota signatures. *Micrococcales* order was upregulated in the irAEs group, whereas *Phocaeicola coprocola* and *Oscillibacter sp* were upregulated in the non-irAEs group (**Figures 1E, F**; **Supplementary Figure 2A**). Given the complex interactions among gut microbes that are critical for maintaining ecological and metabolic homeostasis, we next assessed microbial associations using correlation analysis based on species abundance and variation. The complex interrelationships between the dominant species *Phocaeicola coprocola* in the non-irAEs group and *Phocaeicola plebeius* within the same genus suggest that these two species may play similar roles in irAEs (**Figure 1G**).

### 3.3 Dynamic changes in gut microbiota of irAEs patients

**Figure 2:**
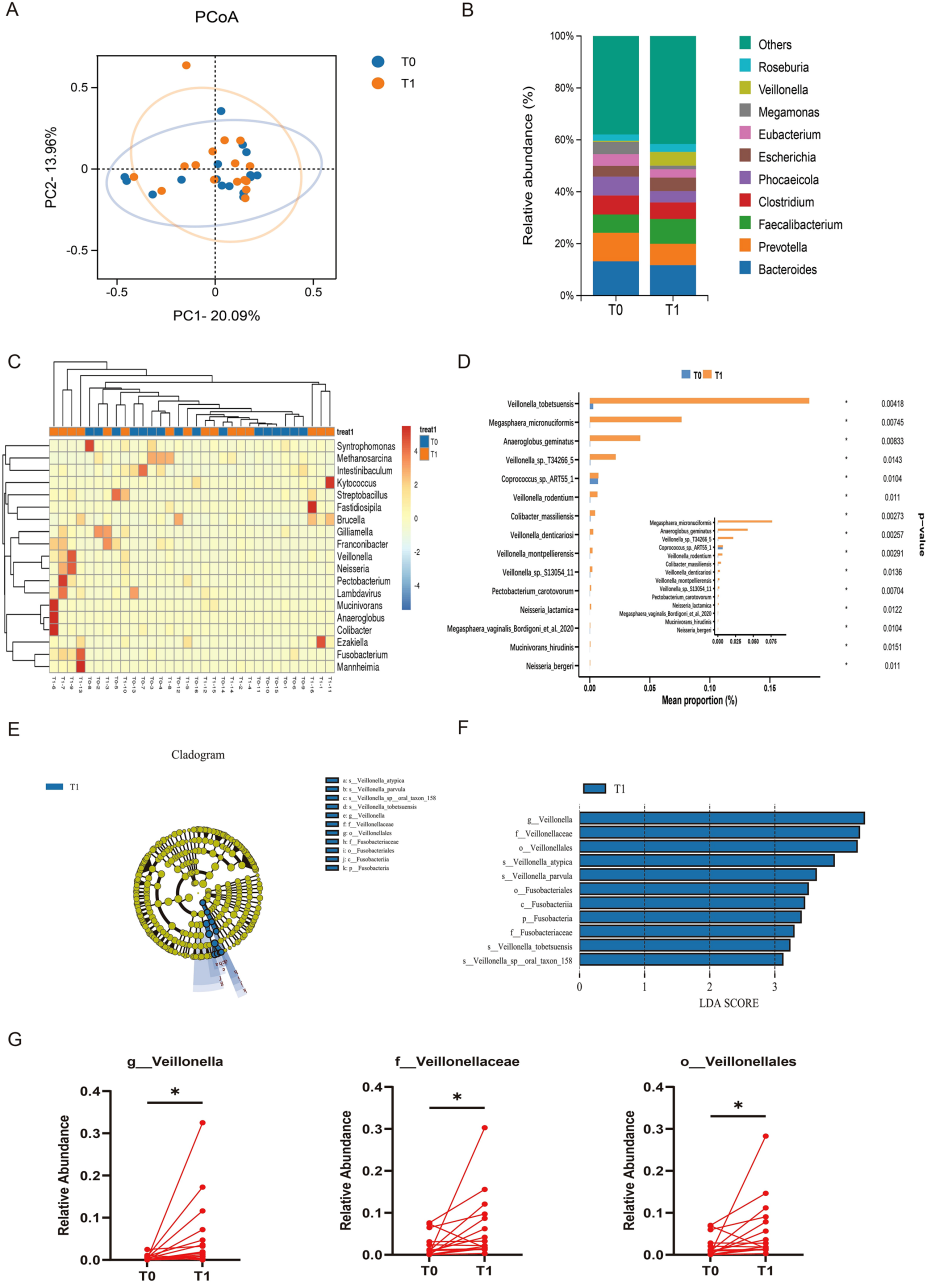
Dynamic changes in gut microbiota of irAEs patients. **(A)** Beta diversity analysis.**(B)** Histogram of gut microbiota relative abundance with the top 10 genera. **(C)** Heatmap of differentially abundant genera. **(D)** Bar chart showing the abundance of differential genera. **(E)** Circular cladogram illustrating taxonomic branching patterns. **(F)** Bar chart of LDA scores for discriminative taxa (LDA ≥ 3). **(G)** Individual variations in the relative abundance of different gut microbiota. *p < 0.05.

We conducted metagenomic analysis on fecal samples from irAEs patients collected at baseline (T0 group) and during irAEs onset (T1 group). Beta diversity analysis at the genus level revealed a separation in the distribution of samples between the two groups, indicating differences in the composition and structure of microbial communities before and during irAEs onset (**Figure 2A**). However, it is regrettable that the result lacks statistically significant support(*P* = 0.601). At the genus level, microbiota composition analysis revealed that the relative abundance of *Veillonella* was higher during irAEs onset, whereas the relative abundance of *Phocaeicola* was lower than that at baseline in irAEs patients (**Figure 2B**). The clustering heatmap further demonstrated pronounced dynamic changes in gut microbiota composition before and after irAEs onset (**Figure 2C**). To identify differential taxa, inter group differential species analysis showed that *Veillonella tobetsuensis*, *Megasphaera micronuciformes*, and *Anaeroglobus geminatus* were enriched in the T1 group (**Figure 2D**). LEfSe analysis further indicated that *Veillonellales*, *Fusobacteria*, and their associated branch strains were upregulated during irAEs (**Figures 2E, F**; **Supplementary Figure 2B**). In addition, the relative abundance of *Veillonella* at the genus level, *Veillonellaceae* at the family level, and *Veillonellales* at the order level showed statistically significant changes before and after irAEs onset, suggesting that these taxa may play potential roles in irAEs progression (**Figure 2G**).

### 3.4 Potential biomarkers of irAEs in baseline gut metabolites

**Figure 3:**
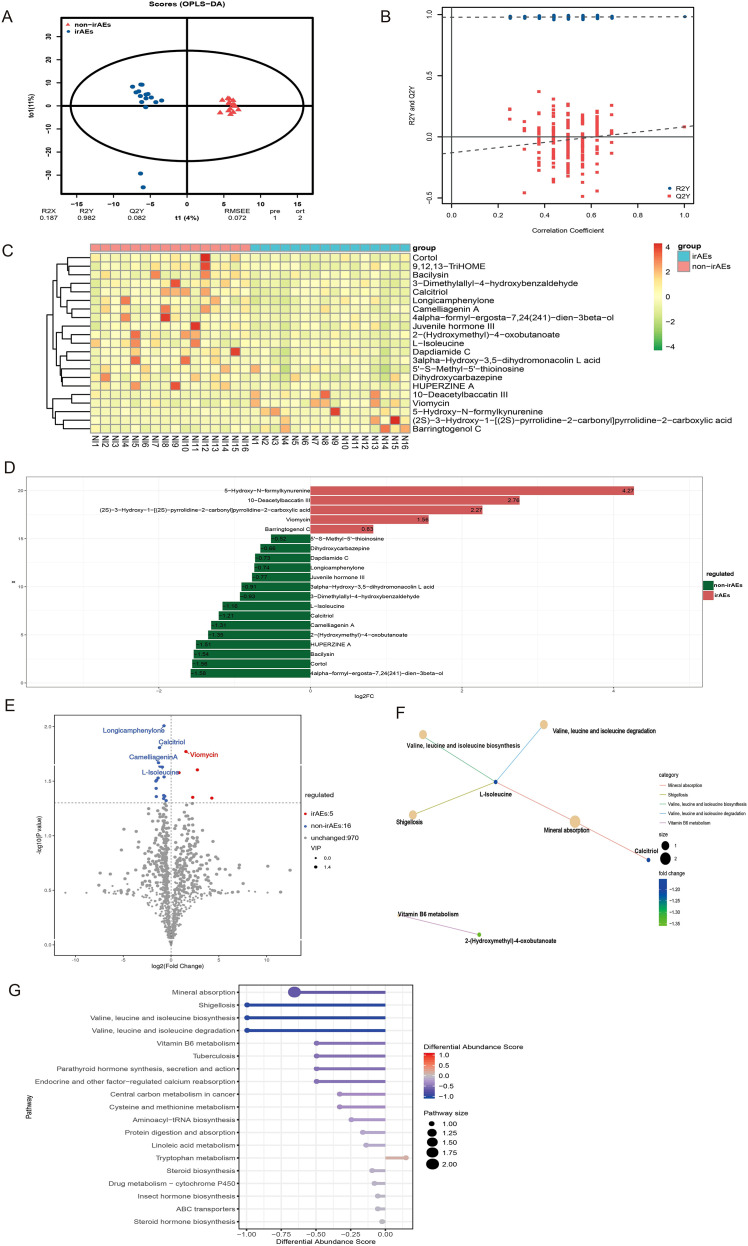
The baseline gut metabolites of patients with/without irAEs. **(A, B)** OPLS-DA shows the separation of metabolic characteristics between irAEs and non-irAEs groups. **(C)** Differential metabolites clustering heatmap. **(D)** Bar chart of differential multiples for the top 20 metabolites. **(E)** Volcano plot highlighting significantly up- and down-regulated metabolites between irAEs and non-irAEs groups. **(F)** Network diagram of KEGG pathway enrichment for the differential metabolites. **(G)** Differential abundance score plot of KEGG pathway for differential metabolites.

Untargeted metabolomics analysis was performed on baseline fecal samples, and 991 metabolites were annotated. OPLS-DA clustering analysis showed clear separation of metabolite profiles between the two groups, indicating significant differences in metabolic characteristics (**Figures 3A, B**). The clustering heatmap of differential metabolites demonstrated varying degrees of enrichment in the non-irAEs group (**Figure 3C**). Further differential analysis identified 21 metabolites, including 16 metabolites upregulated in the non-irAEs group and 5 metabolites upregulated in the irAEs group (**Figures 3D, E**). The metabolites with the most significant statistical differences were viomycin, longicamphenylone, calcitriol, camelliagenin A, and L-isoleucine (**Figure 3E**). Viomycin was upregulated in the irAEs group, whereas longicamphenylone, calcitriol, camelliagenin A, and L-isoleucine were upregulated in the non-irAEs group. These differential metabolites may interact within the host and participate in distinct metabolic pathways. To further interpret these findings from a physiological perspective, we annotated the differential metabolites using the KEGG database and focused on pathways with the largest number of annotated differential metabolites, including mineral absorption, shigellosis, valine, leucine and isoleucine biosynthesis, valine, leucine and isoleucine degradation, and vitamin B6 metabolism. Calcitriol was associated with mineral absorption, while L-isoleucine was linked to multiple metabolic pathways (**Figure 3F**). Compared with the non-irAEs group, the functions of these pathways were downregulated in the irAEs group (**Figure 3G**).

The differential metabolite viomycin was significantly enriched in the irAEs group, whereas longicamphenylone, calcitriol, camelliagenin A, and L-isoleucine showed higher levels in the non-irAEs group, suggesting that these metabolites may have a protective effect against irAEs (**Supplementary Figure 3A**). ROC analysis was conducted for each differential metabolite, and the resulting AUC values further supported their potential utility in predicting irAEs (**Supplementary Figure 3B**). Together, these findings indicate that gut microbiota associated metabolic alterations are closely involved in irAEs, and that specific metabolites and related metabolic pathways may serve as candidate biomarkers for irAEs prediction.

### 3.5 Dynamic changes of gut metabolites in irAEs patients

**Figure 4:**
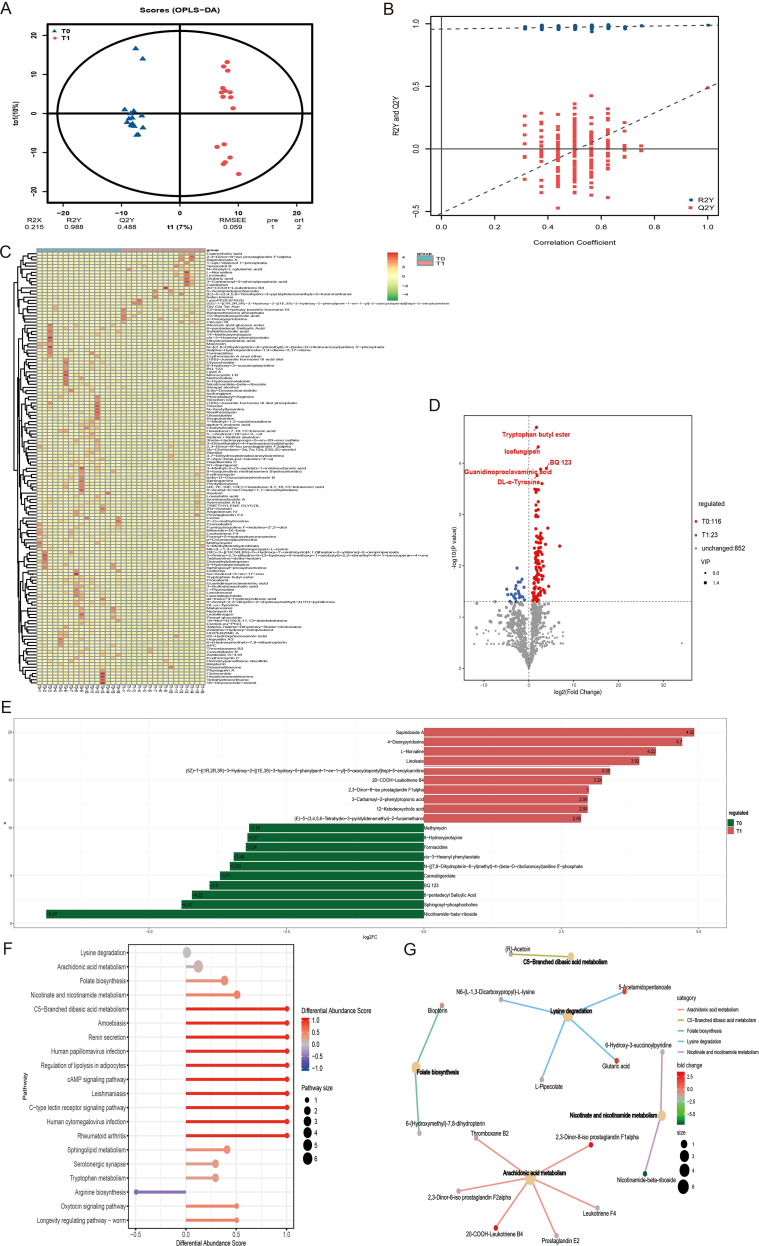
Dynamic changes of gut metabolites in irAEs patients. **(A, B)** OPLS-DA shows the separation of metabolic characteristics between T0 and T1 groups. **(C)** Differential metabolites clustering heatmap. **(D)** Volcano plot highlighting significantly up- and down-regulated metabolites between T0 and T1 groups. **(E)** Bar chart of differential multiples for the top 20 metabolites. **(F)** Differential abundance score plot of KEGG pathway for differential metabolites. **(G)** Network diagram of KEGG pathway enrichment for the differential metabolites.

To investigate changes in gut metabolic signatures during the development of irAEs, we analyzed metabolites from irAEs patients collected at baseline and during irAEs episodes. OPLS-DA clustering analysis of annotated metabolites showed clear separation between the two groups, indicating marked metabolic alterations in the gut during irAEs development (**Figures 4A, B**). The hierarchical clustering heatmap of differential metabolites showed that these metabolites were mainly enriched, to varying degrees, in the T1 group (**Figure 4C**). Further differential analysis identified 139 differential metabolites, including 116 metabolites upregulated in the baseline group (T0 group) and 22 metabolites upregulated during irAEs occurrence (T1 group). The top 20 differential metabolites were selected for visualization, and metabolites with significant statistical differences included tryptophan butylester, BQ 123, DL-o-tyrosine, and nicotinamide-beta-riboside (**Figures 4D, E**). KEGG pathway analysis of the differential metabolites indicated their involvement in multiple physiological pathways, and the functional activities of these pathways were downregulated in the T0 group (**Figure 4F**). The pathways with the highest enrichment of differentially expressed metabolites included lysine degradation, arachidonic acid metabolism, folate biosynthesis, nicotinate and nicotinamide metabolism, and C5-branched dibasic acid metabolism (**Figure 4G**).

The differential metabolites trypsin butylester, BQ 123, DL-o-tyrosine, and nicotinamide-beta-riboside were significantly increased in the T0 group, and their levels decreased with the development of irAEs (**Supplementary Figure 4A**). ROC analysis was performed for each of these differential metabolites, and the resulting AUC values further supported their potential relevance to the development of irAEs (**Supplementary Figure 4B**). These findings indicate that lung cancer patients who exhibit reduced levels of trypsin butylester, BQ 123, DL-o-tyrosine, and nicotinamide-beta-riboside during ICIs treatment may have a higher risk of developing irAEs.

### 3.6 The correlation between baseline differential metabolites and peripheral blood lymphocytes in lung cancer patients receiving ICIs treatment

**Figure 5:**
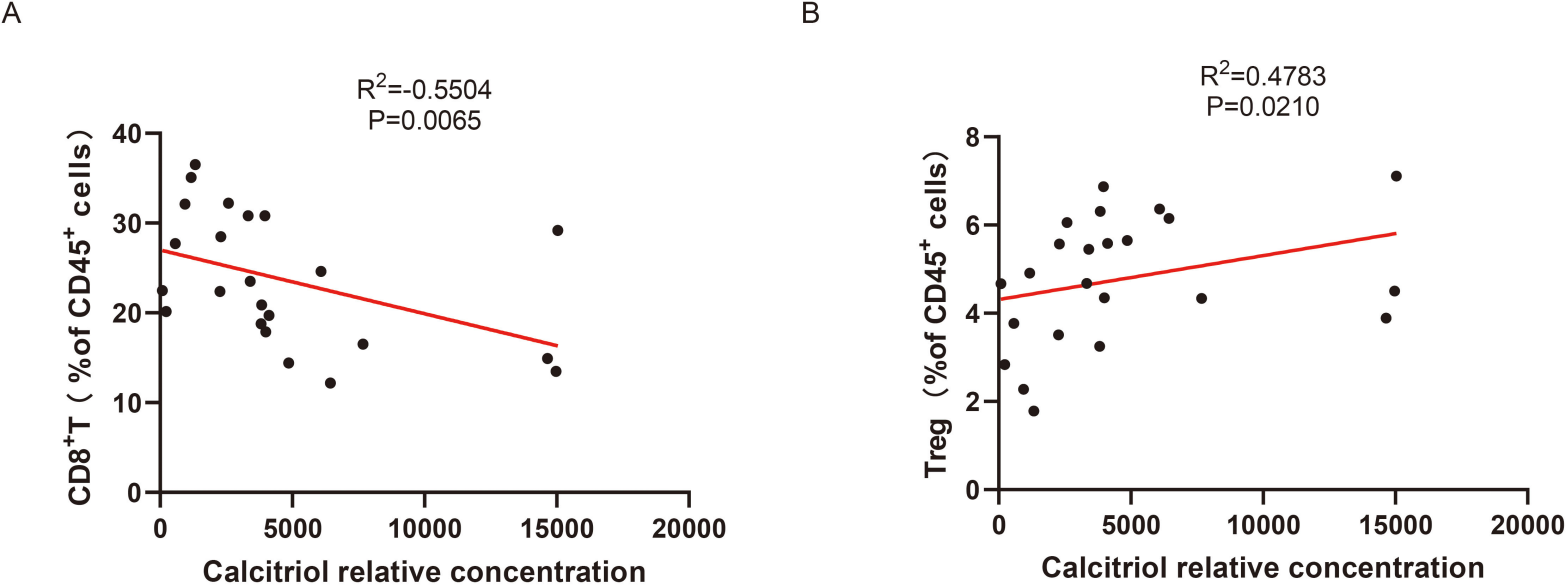
Correlation analysis between baseline differential metabolites and lymphocytes in lung cancer patients receiving ICIs treatment. **(A)** Correlation analysis between CD8^+^T cell ratio and calcertiol content. **(B)** Correlation analysis between Treg cells ratio and calcertiol content.

Multiple studies have reported an association between irAEs and the distribution of lymphocyte subsets. Reduced levels of anti-inflammatory immune cells, such as Treg cells, together with a more pro-inflammatory immune milieu dominated by CD8^+^T cells, may increase susceptibility to irAEs (12, 13). Our patient data also showed an increase in CD8^+^T cells after irAEs (**Supplementary Figure 5**, **Supplementary Table 1**). To investigate the association between baseline irAEs related gut metabolites and baseline lymphocyte profiles, we collected peripheral blood lymphocyte data and performed correlation analysis between metabolites and lymphocyte subsets (**Supplementary Table 2**). We found a statistically significant correlation between elevated host-derived calcitriol levels and a reduced proportion of CD8^+^ T cells as well as an increased proportion of Treg cells, whereas the other four metabolites showed no statistically significant correlations with lymphocyte subsets (**Figure 5**, **Supplementary Figure 6**). These results suggest that patients with higher intestinal calcitriol levels may have established a more immune tolerant state before receiving ICIs treatment, thereby being less susceptible to irAEs. Collectively, host-derived calcitriol may have a potential preventive effect on irAEs.

### 3.7 Multi omics joint analysis reveals the correlation between gut microbiota and metabolites in irAEs

**Figure 6:**
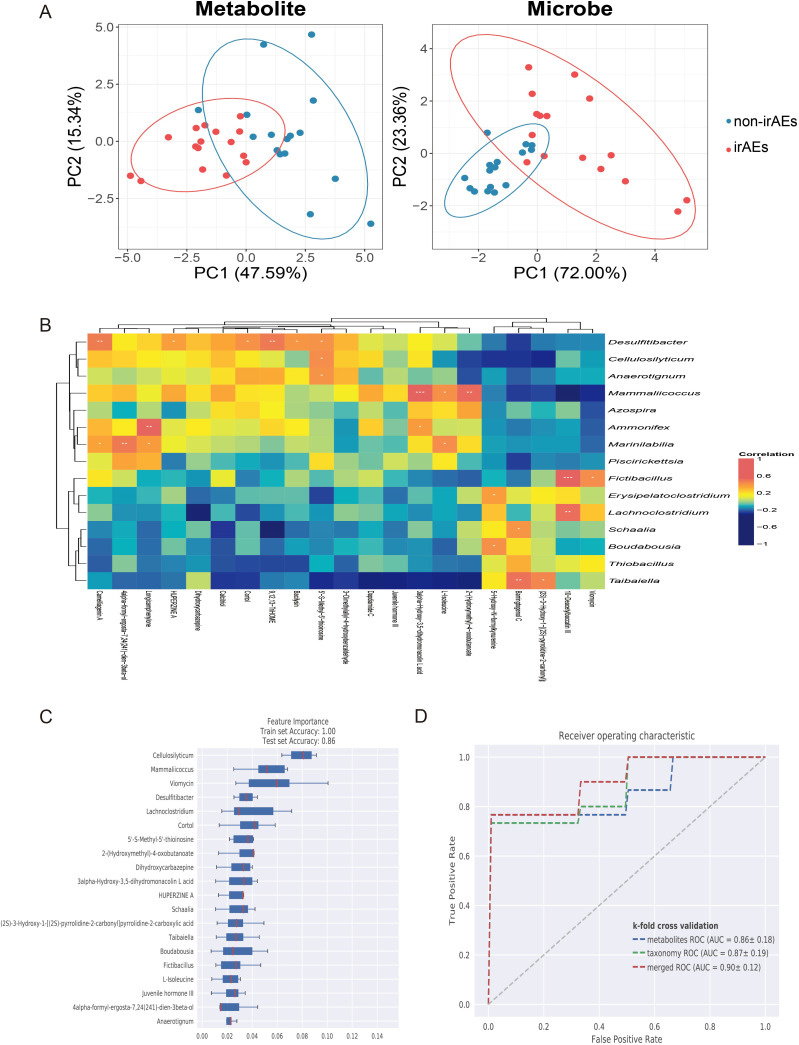
Correlation between gut microbiota and metabolites in irAEs patients. **(A)** PCoA illustrating clear clustering based on metabolomic and microbiome profiles across groups. **(B)** Heatmap displaying correlations between differential metabolite levels and microbial species abundance. **(C)** Feature importance ranking of key microbial and metabolic biomarkers that differentiate irAEs patients using a random forest model. **(D)** ROC curve of random forest model. *p < 0.05, **p < 0.01, ***p < 0.001.

To explore the association between gut microbiota and metabolites, we performed an integrated analysis of metagenomic and metabolomic data. Dimensionality reduction and comparative multi omics analyses revealed significant differences in gut microbiota and metabolite profiles between the irAEs and non-irAEs groups (**Figure 6A**). We also observed significant correlations between gut microbiota derived metabolites and species abundance (**Figure 6B**). For example, camelliagenin A was significantly positively correlated with *Desulfitibacter*, and L-isoleucine was significantly positively correlated with *Mammaricoccus*, indicating complex interactions between microbial communities and metabolic pathways.

We further applied machine learning to construct a random forest model integrating metabolomic and metagenomic data. First, feature importance ranking was used to identify key biomarkers for irAEs prediction. The analysis showed that microbial species, such as *Cellulosilyticum*, and metabolites, such as viomycin, contributed substantially to irAEs prediction in lung cancer (**Figure 6C**). Based on these selected microbial and metabolic features, we developed models using individual omics datasets as well as an integrated multi omics dataset, and compared ROC curves to assess their ability to discriminate between the irAEs and non-irAEs groups. The integrated model achieved an AUC of 0.90, indicating strong discriminative performance in distinguishing irAEs from non-irAEs (**Figure 6D**). Together, these results deepen our understanding of irAEs associated features and highlight the value of multi omics integration for biomarker discovery.

## 4 Discussion

The development of ICIs represents a revolutionary breakthrough in cancer therapy and has markedly improved outcomes for patients with multiple tumor types. However, the clinical use of ICIs is constrained by irAEs (1). IrAEs are off target effects of an overactivated immune system on various normal tissues (14). The incidence of irAEs in patients receiving ICIs treatment can be as high as 50%. IrAEs lack specific target tissues and may involve almost any organ system, and the time to onset is highly variable, with no fixed time point (15). Therefore, given the high incidence, broad spectrum of organ involvement, and unpredictable onset of irAEs, they have become a major challenge in clinical practice. There is an urgent need to develop predictive approaches for irAEs to enable early prevention and timely intervention. Notably, patients who develop irAEs often demonstrate more favorable responses to ICIs, and multiple studies have reported that, compared with patients without irAEs, those who experience irAEs have significantly longer progression free survival and overall survival (16). Especially in non-small cell lung cancer, the overall survival of irAEs patients can be doubled (5). At present, there is no specific treatment strategy for irAEs. In clinical practice, corticosteroids and immunosuppressants are empirically used as first-line therapies, and severe irAEs often require discontinuation of ICIs. However, corticosteroids, immunosuppressants, and interruption of ICIs can substantially compromise the therapeutic efficacy of ICIs (17–20). Because irAEs are treatment related toxicities induced by anti-tumor drugs, their management must also account for the potential impact of therapeutic interventions on tumor control. Therefore, developing new strategies for irAEs management that do not compromise the efficacy of ICIs remains a key challenge.

The human gut microbiota is a complex ecosystem that influences multiple host functions, including metabolism and immune regulation, and its intricate interactions with the immune system have attracted substantial attention (21). With the continued expansion of research on the gut microbiota and its metabolites, accumulating evidence has revealed their beneficial roles in modulating the efficacy of tumor immunotherapy and potentially mitigating treatment related side effects (22, 23). Some bacteria such as *Bifidobacterium* can alleviate irAEs symptoms while synergizing with ICIs (24). Identifying these bacteria and promoting their colonization may help maximize the clinical benefits of ICIs. The gut microbiota represents a highly promising avenue for reducing irAEs, and recent studies investigating the impact of gut microbiota on irAEs have revealed a clear association between the two, potentially opening new avenues for irAEs prevention and management.

A study involving 18000 lung cancer patients receiving ICIs treatment showed that antibiotic use was significantly associated with an increased risk of irAEs, potentially due to reduced gut microbiota diversity and disruption of normal community composition and structure (25). Many studies have confirmed that the composition of gut microbiota differs between patients with and without irAEs (9, 10, 26–28). The gut microbiota is not only associated with the risk of irAEs, but also with the severity level of irAEs. Patients with severe irAEs have higher baseline abundance of *Streptococcus*, *Streptococcus* and *Stenotrophomonas* of the *Pseudomonadota phylum*, while patients with mild irAEs have enrichment of *Faecalibactrium* and unidentified *Lachnospiraceae (*29). The gut microbiota is also involved in organ specificity during the onset of irAEs, and gastrointestinal irAEs have their own gut microbiota characteristics (22, 30). Interestingly, irAEs associated with different classes of ICIs appear to be linked to distinct gut microbiota signatures. Analyses across multiple cohorts receiving different ICIs showed that *Lactobacillus salivarius* and *Fusobacterium mortiferum* were enriched at baseline in patients who did not develop irAEs after treatment with anti-PD - (L) 1 antibodies, whereas Bacteroidetes were enriched in patients who did not develop irAEs after treatment with anti-CTLA-4 antibodies (9).

Our study found that *Faecalibactrium* and *Phocaeicola coprocola* were enriched in the non-irAEs group, whereas *Prevotella* was enriched in the irAEs group. *Faecalibacterium prausnitzii* is a key anti-inflammatory species within *Faecalibacterium* and has been reported to ameliorate ICIs induced colitis while enhancing anti-tumor activity, potentially in association with increased gut microbiota diversity (30). Baseline enrichment of *Phocaeicola plebeius* has been associated with resistance to irAEs in patients with advanced solid tumors receiving ICIs treatment (9). Our network analysis shows that *Phocaeicola coprocola* and *Phocaeicola plebeius* interact with each other and have a strong correlation, suggesting that they may have the same effect in the human body. In a metagenomics sequencing study on the gut microbiota of lung cancer patients receiving anti-PD - (L) 1 antibody therapy, irAEs were associated with higher baseline abundance of *Prevotella copri* (10), and many peptide mimetics with autoimmune potential were identified in *Prevotella copri*. Cross reactivity between self-antigens and microbial antigens may be the basis for the pathogenesis of irAEs.

The gut microbiota is not always stable, but changes with the external environment and physiological state. Changes in the gut microbiota during disease development may affect the outcome of the disease. There are case reports describing the dynamic changes in gut microbiota from onset to remission of ICIs induced colitis. The abundance of *Bacteroidetes* decreased significantly after the occurrence of irAEs and recovered after the resolution of irAEs (31). We observed a similar pattern in our cohort, in which *Bacteroides* decreased during irAEs onset, whereas the abundance of the *Veillonellaceae* family and the *Veillonella* genus within the *Veillonellales* order increased during the onset of irAEs. Notably, the gut microbiota composition 10 days before the onset of immune-related colitis has been reported to resemble that of inflammatory bowel disease, characterized by reduced microbial diversity and increased abundance of *Proteobacteria* and *Veillonella (*32). Among hepatocellular carcinoma patients treated with ICIs, *Prevotella* was enriched in the gut of patients with poor responses to ICIs, whereas *Veillonella* was identified as a predictor in patients who responded well to ICIs (33). While *Veillonella* may synergize with ICIs to enhance anti-tumor immunity, excessive immune activation can also damage normal tissues. Therefore, maintaining *Veillonella* at an appropriate abundance may be important to balance its immunomodulatory effects and minimize the risk of irAEs.

The gut microbiota performs complex and highly active metabolic functions in the intestine, providing energy and nutrients required for microbial growth and reproduction while generating a wide range of metabolites. These metabolites may influence irAEs by modulating immune cell activity and inflammatory mediators. Sequencing studies in lung cancer patients receiving anti-PD-1 have shown that the abundance of major butyrate producing bacteria was lower in patients with irAEs than in those without irAEs (22). There is also evidence to support a decrease in butyrate levels at baseline in patients with irAEs (34, 35). Supplementing butyrate or the recolonization of *Prevotella loescheii*, which can produce short-chain fatty acids (SCFAs), can alleviate ICIs induced cardiac toxicity by downregulating the production of TNF and IL-1β (36). These findings suggest that gut microbiota derived SCFAs may play a preventive role against irAEs. In addition, supplementation with indole-3-carboxaldehyde, a metabolite produced through microbial tryptophan metabolism, has been reported to prevent ICIs induced colitis without compromising anti-tumor activity (37).

Our study found that, at baseline, viomycin was positively correlated with irAEs, whereas calcitriol and L-isoleucine were negatively correlated with irAEs. Calcitriol is the active form of vitamin D and not only regulates calcium and phosphorus metabolism, but also exerts important immunoregulatory effects, particularly in the induction and maintenance of Treg cell function (38). Calcitriol can also induce macrophage polarization from M1 phenotype to M2 phenotype, thereby reducing inflammation (39). Similarly, our results suggest a possible correlation between intestinal calcitriol levels and the proportions of CD8^+^ T cells and Treg cells, indicating that calcitriol may contribute to the prevention of irAEs by fostering an immune-tolerant environment and mitigating over-activation of the immune system by ICIs. The role of calcitriol in antitumor immunity has not yet been fully elucidated, and the present study provides correlational evidence that cannot establish causality. In the era of immunotherapy, a key scientific challenge lies in identifying a precise regulatory balance between mitigating irAEs and sustaining antitumor immunity. We look forward to future prospective studies that will clarify the relationship between calcitriol and antitumor immunity, ultimately enabling its concentration to be maintained within a precise range that maximizes therapeutic benefit while minimizing toxicity. Administration of L-isoleucine can alleviate dextran sulfate sodium induced colitis in rats by reducing pro-inflammatory cytokines (40), suggesting that L-isoleucine can also regulate immune function and affect irAEs. There is research confirming that serum valine and isoleucine can predict the overall survival of patients receiving ICIs (41), indicating that the metabolism of valine and isoleucine in the body can affect the immune environment, especially in patients receiving ICIs. It can be inferred that valine and isoleucine may also be associated with irAEs. Functional pathway analysis of fecal metagenomic data from patients with ICIs induced colitis showed that gut microbiota profiles characterized by impaired vitamin B biosynthesis were associated with an increased risk of irAEs (42). Another set of data suggests that several microbial metabolic pathways, including vitamin B synthesis, are inhibited in patients with severe irAEs (35). We observed that metabolites downregulated in the irAEs group were primarily enriched in pathways such as valine, leucine and isoleucine metabolism and vitamin B6 metabolism. Dynamic monitoring further indicated that metabolites downregulated during irAEs progression were mainly enriched in pathways including lysine degradation, arachidonic acid metabolism, folate biosynthesis, nicotinate and nicotinamide metabolism, and C5-branched dibasic acid metabolism. However, the current evidence remains associative, and does not constitute direct proof that these pathways actively regulate immune processes or directly cause irAEs. Multiple studies have also supported links between these pathways and human immunity, particularly anti-tumor immunity (43–45). At present, further studies are needed to elucidate the underlying mechanisms, and in the future, regulating these metabolic pathways may provide a potential strategy to prevent the occurrence and progression of irAEs.

Some studies have established classification models for predicting irAEs based on differential gut microbiota (9, 27), which have shown excellent performance in predicting the occurrence of irAEs (AUC = 0.88). Meanwhile, other classification models such as *in vivo* metabolites and immune features also have potential roles in predicting irAEs (46, 47). Therefore, we performed an integrated analysis of baseline fecal metagenomic and metabolomic data to develop a machine learning based random forest classification model, and the model showed strong discriminative performance for predicting irAEs occurrence, with an AUC of 0.90. To facilitate the translation of research findings into clinical practice, the following standardized workflow—”Baseline Screening – Risk Assessment – Personalized Intervention and Monitoring”—could be established after further validation in larger cohorts: 1. Baseline Screening: Fecal samples (for metagenomic/metabolomic profiling) and peripheral blood samples (for lymphocyte subset analysis by flow cytometry) are collected within one week prior to treatment initiation. 2.Risk Assessment: The obtained data are entered into a multi-omics prediction model to stratify patients into risk categories based on biomarker profiles. 3.Intervention and Monitoring: For High-risk patients, prophylactic intervention (e.g., supplementation with safe-dose calcitriol or microbiota modulators) is initiated before treatment, with dynamic monitoring of indicators every two weeks and adjustment of the strategy as needed during therapy. For Intermediate-risk patients, standard treatment is administered, with core biomarkers reevaluated every four weeks; intervention is promptly implemented if abnormalities are detected. For Low-risk patients, standard treatment and routine follow-up are applied. Nevertheless, such predictive models remain at an early exploratory stage and require further validation and robust evidence before they can be translated into clinical practice.

Here, we outline the clinical challenges posed by irAEs and consider their potential links to the gut microbiota and metabolites. We identified characteristic gut microbiota and metabolite features associated with irAEs, highlighted the dynamic changes in gut microbiota and metabolites during irAEs development, and constructed a model to predict irAEs occurrence in lung cancer patients receiving ICIs treatment. We acknowledge several limitations. First, due to strict data quality control and the long-term follow-up required by our study design, the sample size was relatively small. This study did not perform stratified analysis according to lung cancer histological subtypes; however, existing evidence indicates no clear association between lung cancer subtypes and the differential microbial taxa/metabolites identified here. Future studies could include larger cohorts of patients with different subtypes to conduct stratified analyses and validate the generalizability of the findings. Second, these findings require further validation in an independent validation set to confirm robustness, and the prediction model in particular requires additional cohorts to verify the stability of its performance. In addition, the roles and mechanisms of the differential metabolites in regulating immune cell function warrant further investigation.

## Data Availability

The original contributions presented in the study are included in the **Supplementary Material**, further inquiries can be directed to the corresponding authors.

## Supplementary material

Supplementary Figure 1 Flowchart of Participant Enrollment.

Supplementary Figure 2 MaAsLin2 Analysis of Differential Gut Microbes. **(A)** Differential gut microbiota between Patients with/without irAEs. **(B)** Differential gut microbiota in irAEs patients before and after onset.

Supplementary Figure 3 Diagnostic potential of differential metabolites in patients with/without irAEs. **(A)** Violin plot of differential metabolites. **(B)** ROC plot and box plot of differential metabolites.

Supplementary Figure 4 Diagnostic potential of differential metabolites in irAEs patients before and after onset. **(A)** Individual variations in relative abundance of differential metabolites. **(B)** ROC plot and box plot of differential metabolites.

Supplementary Figure 5 Individual changes in the proportion of lymphocyte before and after irAEs occurrence. **(A)** Individual changes in the proportion of CD8+T cells before and after irAEs occurrence. **(B)** Individual changes in the proportion of Treg cells before and after irAEs occurrence.

Supplementary Figure 6 Correlation analysis between baseline differential metabolites and lymphocytes in lung cancer patients receiving ICIs treatment. **(A)** Correlation analysis between CD8+T cell ratio and metabolites content. **(B)** Correlation analysis between Treg cells ratio and metabolites content.
