# Supplementary figures and images for: Dynamic changes in gut microbiota and metabolites in advanced lung cancer patients with immune-related adverse events
Source: Front Immunol. 2026 Apr 16;17:1731931. doi: 10.3389/fimmu.2026.1731931 (PMC13130655; doi:10.3389/fimmu.2026.1731931)

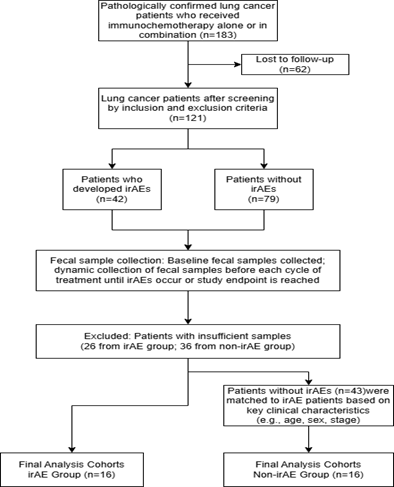

Supplement: Supplementary Figure 1 — Flowchart of Participant Enrollment. [file Image1.tif]

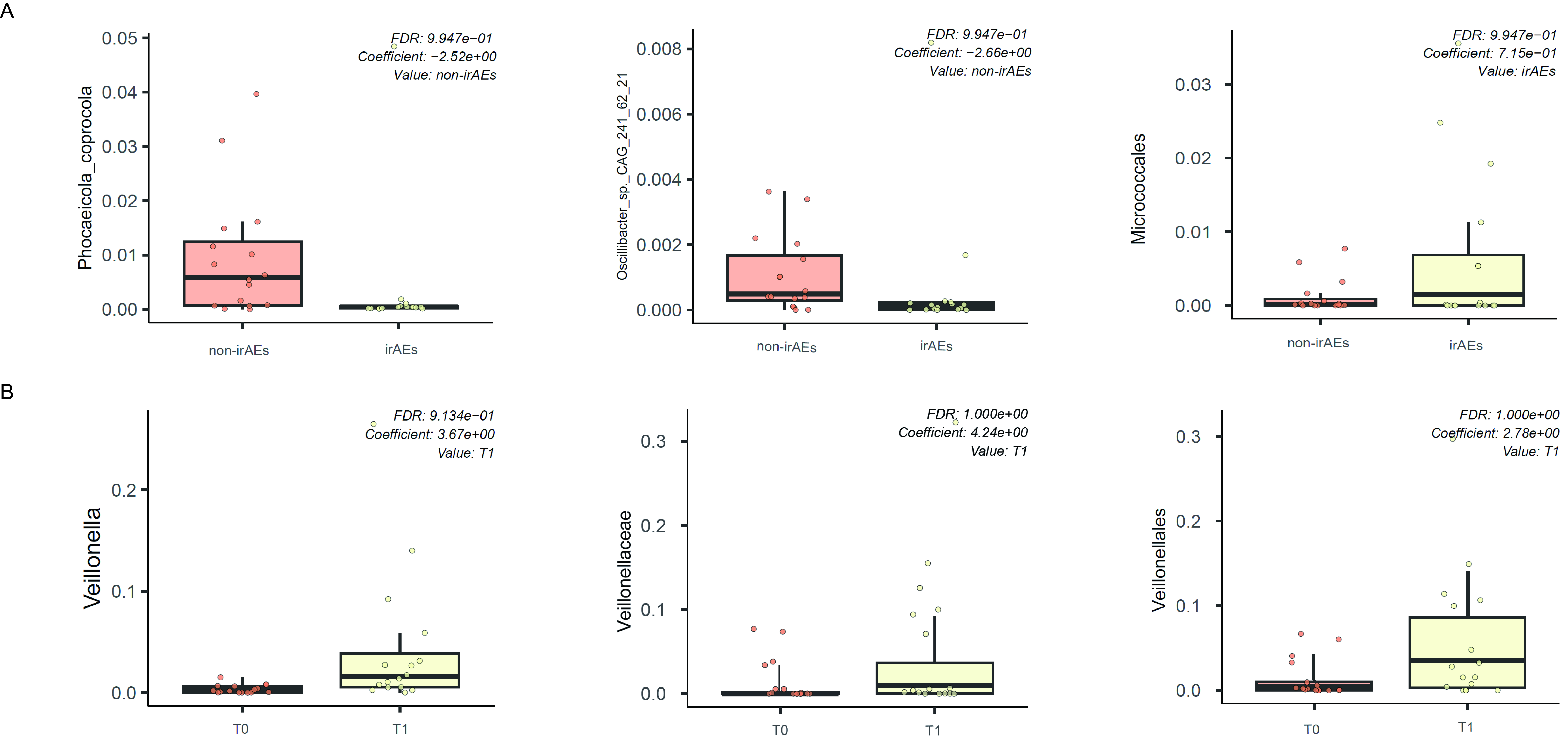

Supplement: Supplementary Figure 2 — MaAsLin2 Analysis of Differential Gut Microbes. (A) Differential gut microbiota between Patients with/without irAEs. (B) Differential gut microbiota in irAEs patients before and after onset. [file Image2.jpg]

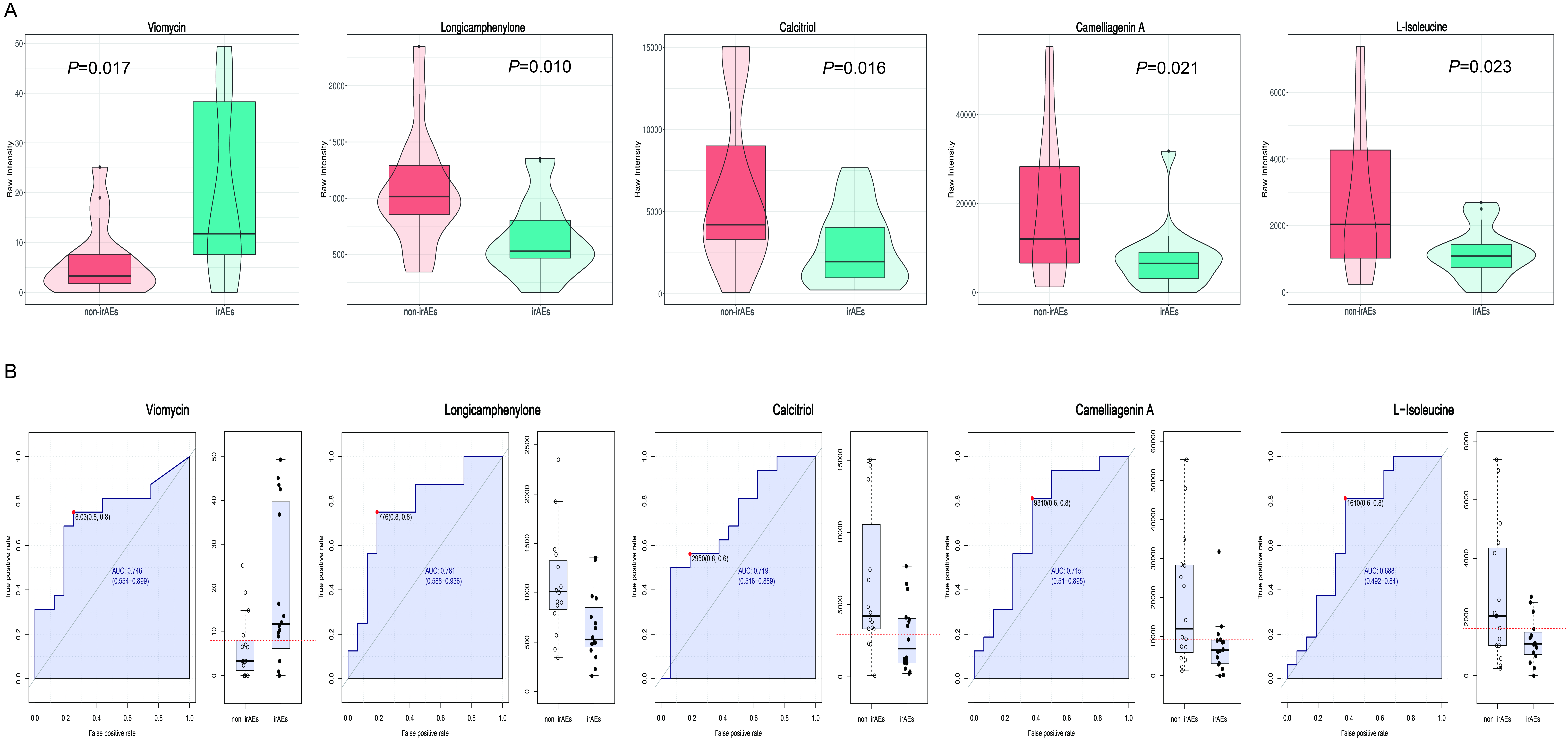

Supplement: Supplementary Figure 3 — Diagnostic potential of differential metabolites in patients with/without irAEs. (A) Violin plot of differential metabolites. (B) ROC plot and box plot of differential metabolites. [file Image3.jpg]

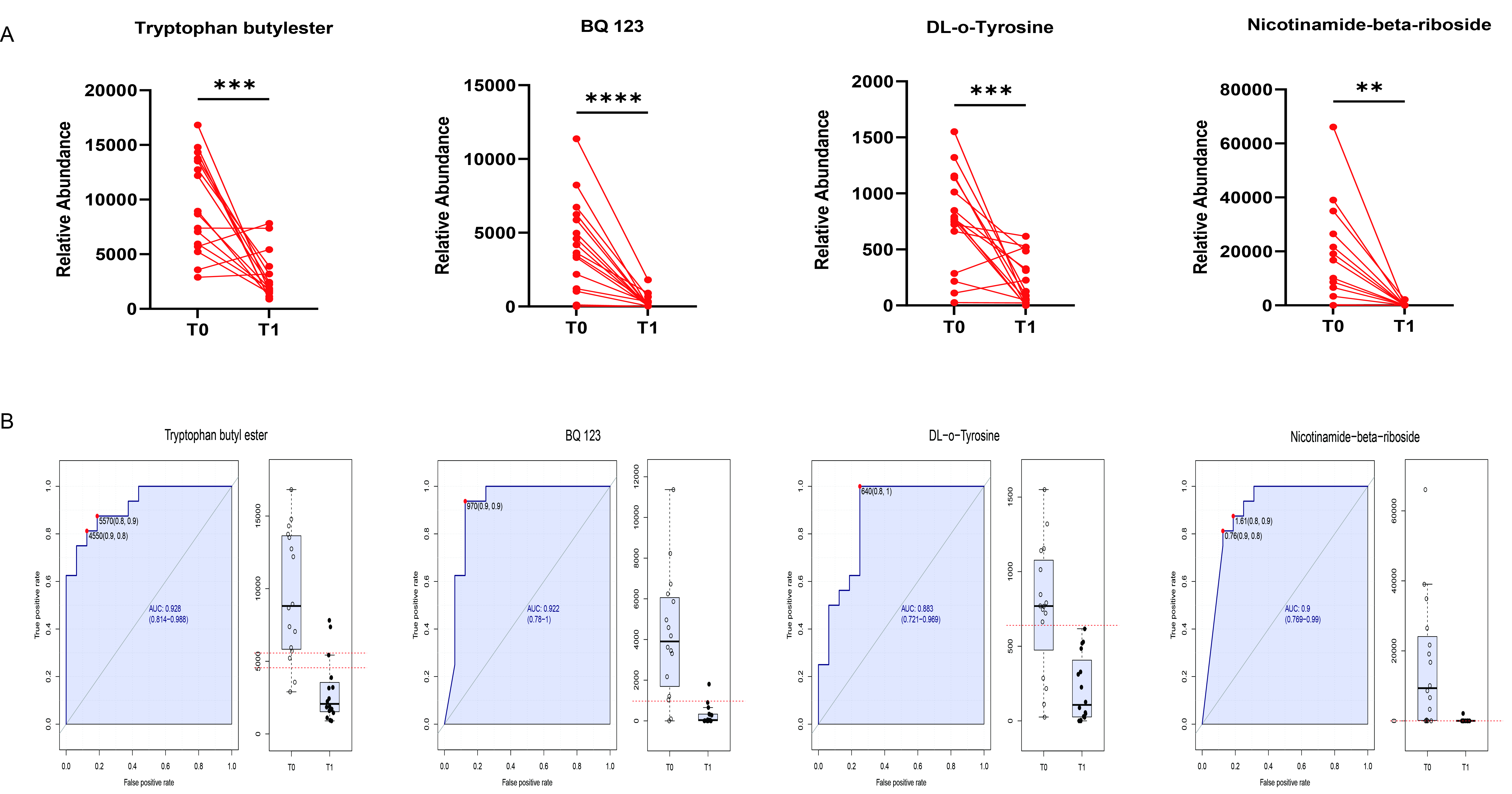

Supplement: Supplementary Figure 4 — Diagnostic potential of differential metabolites in irAEs patients before and after onset. (A) Individual variations in relative abundance of differential metabolites. (B) ROC plot and box plot of differential metabolites. [file Image4.jpg]

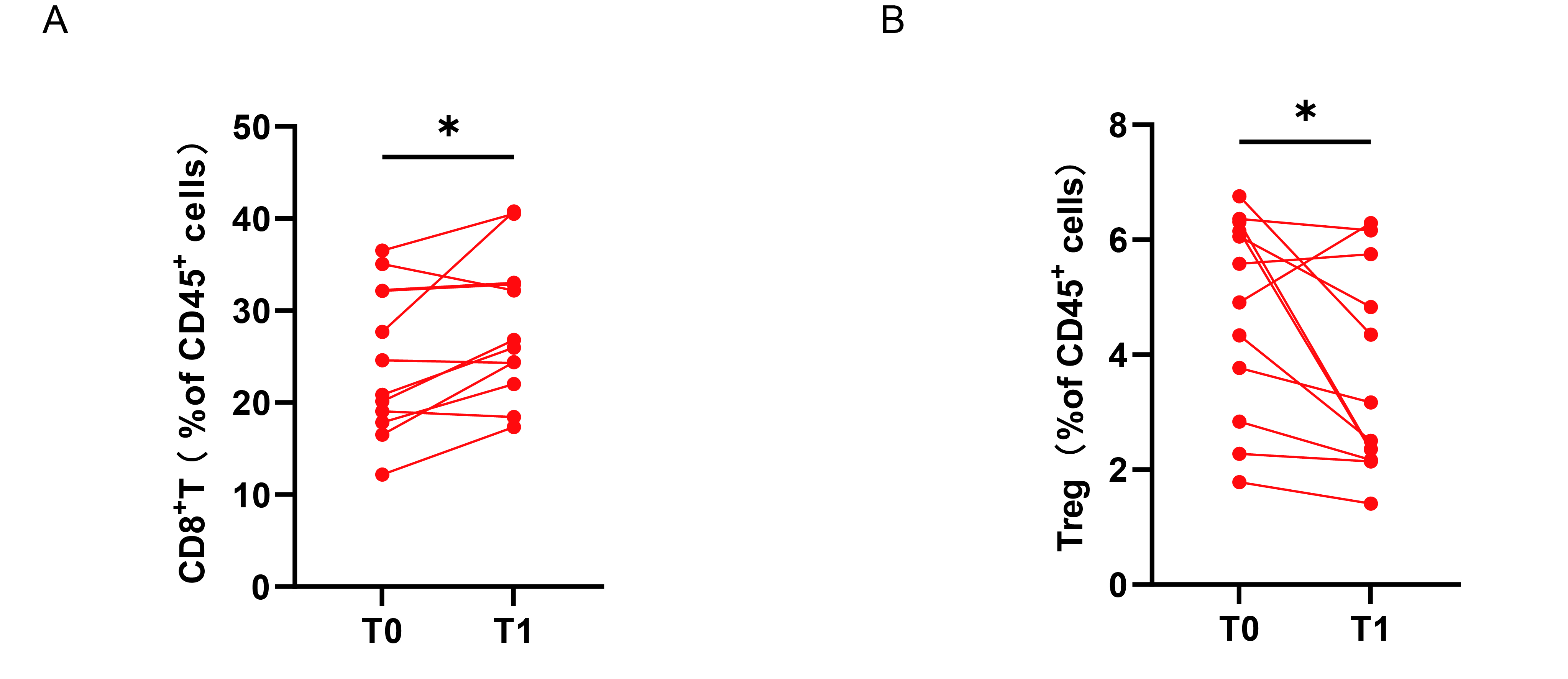

Supplement: Supplementary Figure 5 — Individual changes in the proportion of lymphocyte before and after irAEs occurrence. (A) Individual changes in the proportion of CD8+T cells before and after irAEs occurrence. (B) Individual changes in the proportion of Treg cells before and after irAEs occurrence. [file Image5.jpg]

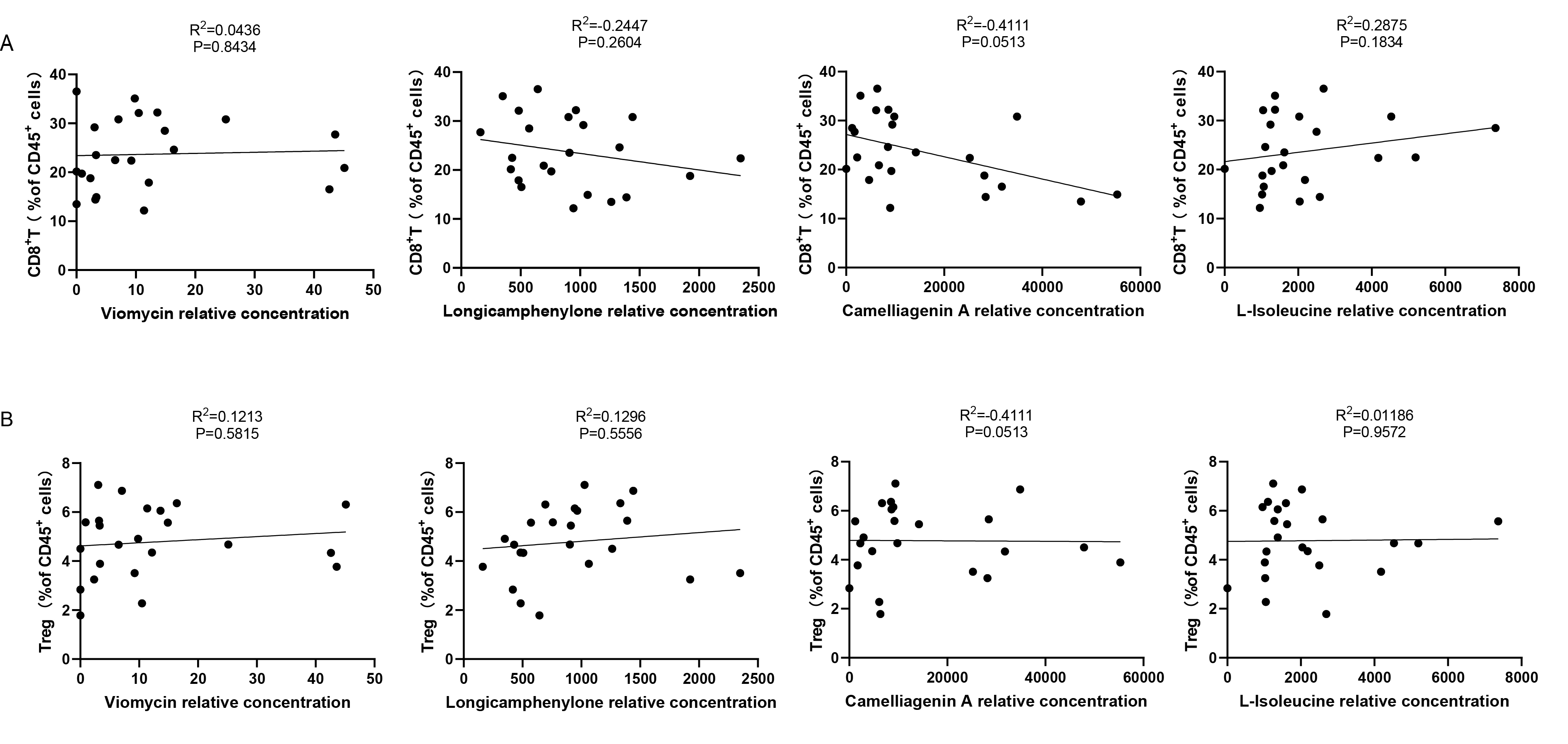

Supplement: Supplementary Figure 6 — Correlation analysis between baseline differential metabolites and lymphocytes in lung cancer patients receiving ICIs treatment. (A) Correlation analysis between CD8+T cell ratio and metabolites content. (B) Correlation analysis between Treg cells ratio and metabolites content. [file Image6.jpg]
