# Supplementary material for: Dynamic changes in gut microbiota and metabolites in advanced lung cancer patients with immune-related adverse events
Source: Front Immunol. 2026 Apr 16;17:1731931. doi: 10.3389/fimmu.2026.1731931 (PMC13130655; doi:10.3389/fimmu.2026.1731931)
Supplement: Supplementary file 7 [file Table1.docx]

| Patient number | Baseline-CD8^+^T(%) | irAEs-  CD8^+^T(%) | Baseline-  Treg(%) | irAEs-  Treg(%) |
| --- | --- | --- | --- | --- |
| non-irAEs-1 | 22.47 | - | 4.67 | - |
| non-irAEs-2 | 30.82 | - | 6.87 | - |
| non-irAEs-3 | 23.50 | - | 5.45 | - |
| non-irAEs-4 | 22.37 | - | 3.51 | - |
| non-irAEs-7 | 30.83 | - | 4.68 | - |
| non-irAEs-8 | 14.90 | - | 3.89 | - |
| non-irAEs-10 | 29.17 | - | 7.11 | - |
| non-irAEs-11 | 28.47 | - | 5.57 | - |
| non-irAEs-12 | 13.47 | - | 4.50 | - |
| non-irAEs-13 | 18.77 | - | 3.25 | - |
| non-irAEs-16 | 14.40 | - | 5.65 | - |
| irAEs-1 | 27.70 | 40.77 | 3.77 | 3.17 |
| irAEs-2 | 32.13 | 32.86 | 2.28 | 2.14 |
| irAEs-3 | 35.07 | 32.17 | 4.91 | 6.29 |
| irAEs-5 | 17.87 | 22.00 | 6.76 | 4.35 |
| irAEs-6 | 19.07 | 18.43 | 5.58 | 5.75 |
| irAEs-7 | 20.87 | 25.97 | 6.31 | 2.36 |
| irAEs-8 | 16.53 | 24.40 | 4.34 | 2.50 |
| irAEs-9 | 36.53 | 40.50 | 1.78 | 1.41 |
| irAEs-10 | 24.60 | 24.30 | 6.36 | 6.16 |
| irAEs-11 | 32.23 | 33.03 | 6.06 | 4.83 |
| irAEs-12 | 12.18 | 17.37 | 6.15 | 2.34 |
| irAEs-14 | 20.14 | 26.83 | 2.84 | 2.17 |

**Supplementary Table 1** Patient lymphocyte ratio data.
