# Supplementary material for: Dynamic changes in gut microbiota and metabolites in advanced lung cancer patients with immune-related adverse events
Source: Front Immunol. 2026 Apr 16;17:1731931. doi: 10.3389/fimmu.2026.1731931 (PMC13130655; doi:10.3389/fimmu.2026.1731931)
Supplement: Supplementary file 8 [file Table2.docx]

**Supplementary Table 2** Patient baseline differential metabolite content.

| Patient number | Viomycin | Calcitriol | Camel  liagenin A | L-isole  ucine | Longicam  phenylone |
| --- | --- | --- | --- | --- | --- |
| non-irAEs-1 | 6.508572 | 87.9288 | 2242.434 | 5193.999 | 428.3301 |
| non-irAEs-2 | 7.05865 | 3961.252 | 34839.03 | 2030.012 | 1440.508 |
| non-irAEs-3 | 3.296677 | 3400.49 | 14221.62 | 1623.84 | 910.5872 |
| non-irAEs-4 | 9.222544 | 2259.013 | 25211.32 | 4178.64 | 2348.634 |
| non-irAEs-7 | 25.14758 | 3328.004 | 9823.808 | 4533.176 | 901.7104 |
| non-irAEs-8 | 3.335046 | 14651.1 | 55276.66 | 1021.303 | 1063.386 |
| non-irAEs-10 | 3.035346 | 15040.75 | 9418.672 | 1247.699 | 1026.978 |
| non-irAEs-11 | 14.88532 | 2291.801 | 1212.037 | 7367.109 | 571.416 |
| non-irAEs-12 | 1.78E-09 | 14969.47 | 47899.64 | 2044.364 | 1261.343 |
| non-irAEs-13 | 2.345296 | 3819.458 | 28186.02 | 1030.922 | 1924.325 |
| non-irAEs-16 | 3.178853 | 4855.807 | 28455.21 | 2588.77 | 1389.226 |
| irAEs-1 | 43.55786 | 566.9615 | 1716.898 | 2500.941 | 161.5896 |
| irAEs-2 | 10.49099 | 940.5046 | 6119 | 1048.24 | 483.609 |
| irAEs-3 | 9.817178 | 1174.2 | 2912.918 | 1371.151 | 348.947 |
| irAEs-5 | 12.18501 | 3989.933 | 4691.205 | 2183.014 | 483.1618 |
| irAEs-6 | 0.915935 | 4116.791 | 9206.565 | 1278.331 | 757.6455 |
| irAEs-7 | 45.11127 | 3843.419 | 6680.628 | 1593.137 | 693.0636 |
| irAEs-8 | 42.59161 | 7667.408 | 31766.48 | 1065.556 | 506.1769 |
| irAEs-9 | 1.78E-09 | 1331.976 | 6351.808 | 2691.985 | 643.3835 |
| irAEs-10 | 16.40707 | 6083.503 | 8503.617 | 1104.341 | 1331.528 |
| irAEs-11 | 13.62827 | 2586.915 | 8606.226 | 1369.392 | 964.6933 |
| irAEs-12 | 11.38547 | 6440.914 | 8984.807 | 959.5187 | 944.3433 |
| irAEs-14 | 1.78E-09 | 232.3579 | 1.78E-09 | 1.78E-09 | 417.7624 |
